# Descending Necrotizing Mediastinitis as a Deadly Complication Following Laparoscopic Sleeve Gastrectomy: A Case Report

**DOI:** 10.70352/scrj.cr.25-0213

**Published:** 2025-06-21

**Authors:** Kengo Kadoya, Kotaro Wakamatsu

**Affiliations:** Department of Surgery, Toho University Sakura Medical Center, Sakura, Chiba, Japan

**Keywords:** laparoscopic sleeve gastrectomy, descending necrotizing mediastinitis, obesity, piriform fossa injury

## Abstract

**INTRODUCTION:**

Laparoscopic sleeve gastrectomy is a standard and safe surgical procedure for patients with morbid obesity. A potential complication is piriform fossa injury, which can occur during calibration tube insertion. We encountered a case of descending necrotizing mediastinitis, a serious and progressive infection originating from a piriform fossa injury that was potentially exacerbated by diabetes.

**CASE PRESENTATION:**

A female patient with morbid obesity (body mass index, 41 kg/m^2^) and a heavy smoking habit underwent laparoscopic sleeve gastrectomy and was discharged without any immediate postoperative complications. Six weeks later, the patient presented with severe chest and back pain that required continuous fentanyl injection for pain management. Initially, staple line leakage, commonly observed after sleeve gastrectomy, was suspected. However, her condition progressively worsened, and she was admitted to the ICU. An enhanced abdominal CT scan extending to the cervical region revealed a cervical abscess extending to the lower mediastinal region. The patient was diagnosed with descending necrotizing mediastinitis. Given the rarity of this disease, we hypothesized that her smoking habits exacerbated the piriform fossa injury caused by the calibration tube used during gastrectomy. Following the diagnosis, emergency abscess drainage surgery was performed, and the patient was successfully treated.

**CONCLUSIONS:**

This is the 1st reported case of descending necrotizing mediastinitis as a fatal complication of laparoscopic sleeve gastrectomy, which was identified and treated successfully owing to the timely and expanded use of an enhanced CT scan that included the cervical region. Traditionally, the cervical area has not been routinely examined when diagnosing complications following abdominal surgery, underscoring the importance of a comprehensive imaging approach from the neck to the abdomen to detect complications after sleeve gastrectomy.

## Abbreviations

BMI
: body mass index
COPD
: chronic obstructive pulmonary disease
CPR
: cardiopulmonary resuscitation
DNM
: descending necrotizing mediastinitis
EGJ
: esophagogastric junction
ICU
: intensive care unit
LSG
: laparoscopic sleeve gastrectomy

## INTRODUCTION

LSG is the most popular bariatric and metabolic surgery and is known for its safety and relatively low complication rates. The most common complication is staple line leakage. However, we previously reported a rare complication of piriform fossa injury caused by calibration tube insertion.^1)^

DNM is a serious and progressive infection that can result from odontogenic, pharyngeal, or cervical infections that disseminate rapidly to the mediastinum. It is a life-threatening condition with a high mortality rate (10%–40%) due to sepsis and organ failure if not diagnosed and treated promptly.^2)^

Here, we present the 1st reported case of DNM following LSG, possibly triggered by a calibration tube injury and pharyngeal infection.

## CASE PRESENTATION

**Fig. 1:**
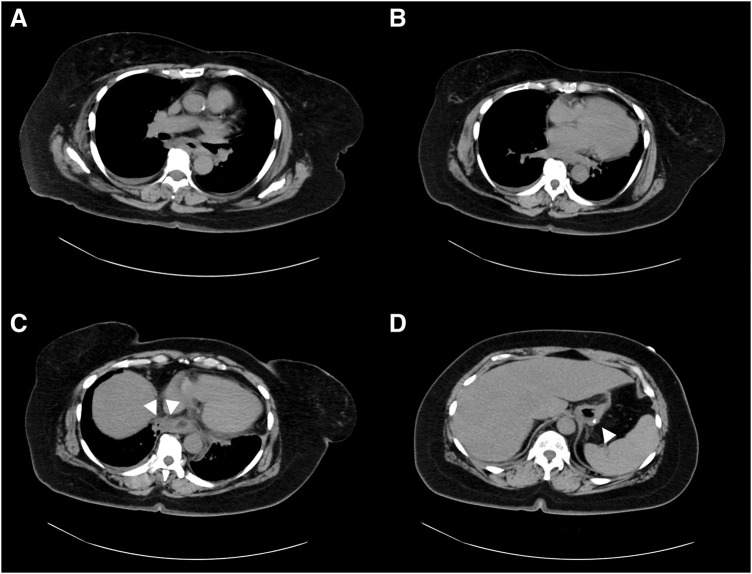
CT scan serial transverse planes from tracheal bifurcation to spleen at admission. (**A**) At the tracheal bifurcation level, no obvious abnormalities were noted. (**B**) At the right atrium level, no apparent abnormalities were noted. (**C**) At the EGJ level, the esophageal wall was slightly thickened circumferentially, and a small amount of fluid collection was noted on the right side of the esophagus (white triangles). (**D**) The upper portion of the linear staple was visible in the spleen. No free air or fluid was collected (white triangle). EGJ, esophagogastric junction

**Fig. 2:**
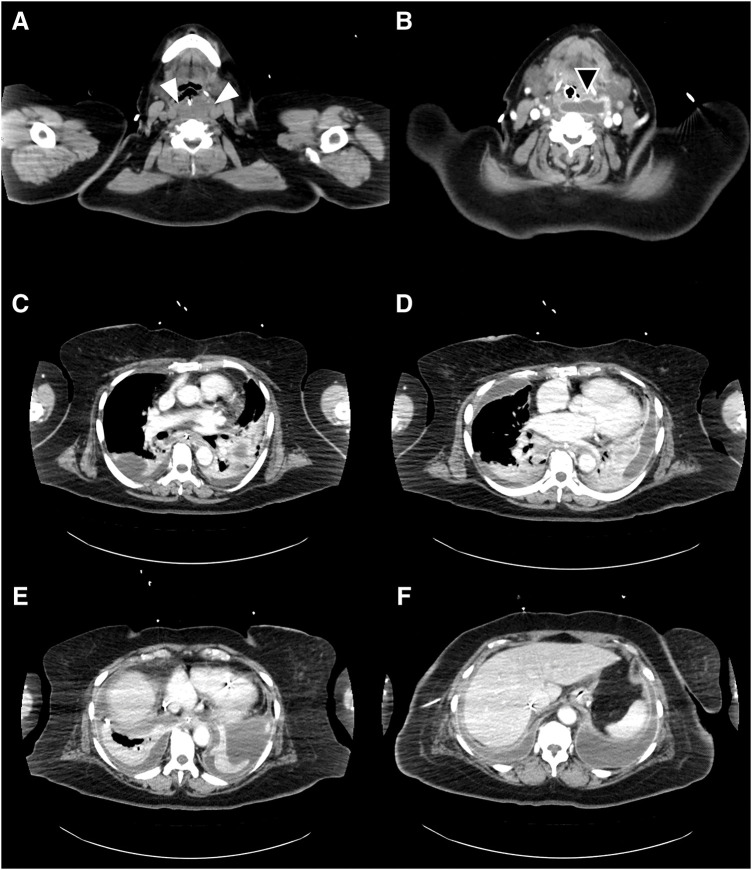
Enhanced CT scan serial transverse planes from the cervical region to the spleen before emergency surgery. (**A**) At the lower pharyngeal level, a low-density mass is noted (white triangles). (**B**) At the cervical esophageal level, an abscess with an enhanced capsule was noted (black triangle). (**C**) At the tracheal bifurcation level, pleural effusion is noted in both thoracic cavities. (**D**) Pleural effusion was noted on the left side of the thoracic cavity at the right atrial level. (**E**) At the EGJ level, massive pleural effusion causing retraction of the inferior lung lobes was noted in both thoracic cavities. (**F**) In the upper portion of the spleen, the left side of the thoracic cavity appeared continuous with a linear stapler. EGJ, esophagogastric junction

A 52-year-old Japanese woman with morbid obesity (BMI 40.8 kg/m^2^) underwent LSG to reduce her excessive weight. She had a long history of obesity-related comorbidities including Type 2 diabetes (hemoglobin A1c 7.0%), hypertension, COPD, knee arthropathy, and asthma. As a standard surgical safety measure, she was required to quit smoking 2 months preoperatively. The patient was discharged on postoperative day 4 without any surgical complications. However, she resumed smoking immediately after discharge and began to experience discomfort in the neck region. Six weeks after discharge, she urgently presented to our emergency care ward with acute, severe chest and back pain, requiring continuous intravenous fentanyl for pain management. Initially, LSG staple line leakage was suspected, although CT did not provide a definitive conclusion (**Fig. 1**). The patient was hospitalized, and a gastric tube was inserted to decompress the internal pressure of the stomach. Antibiotics were concurrently administered. Her respiratory and cardiac conditions progressively worsened, necessitating ICU management for 10 days after admission. On the 11th day of hospitalization, she experienced cardiopulmonary arrest and underwent immediate CPR, which was successful despite drug-resistant ventricular tachycardia. Consequently, a temporary pacemaker was installed for cardiac stabilization. Simultaneously, endotracheal intubation was performed for respiratory management. The diagnosis was reassessed with an enhanced CT scan after stabilizing her cardiopulmonary condition, revealing massive pleural effusions in both thoracic cavities; however, the origin of the fluid collection remained unidentified on standard transverse plane CT scans (**Fig. 2**).

**Fig. 3:**
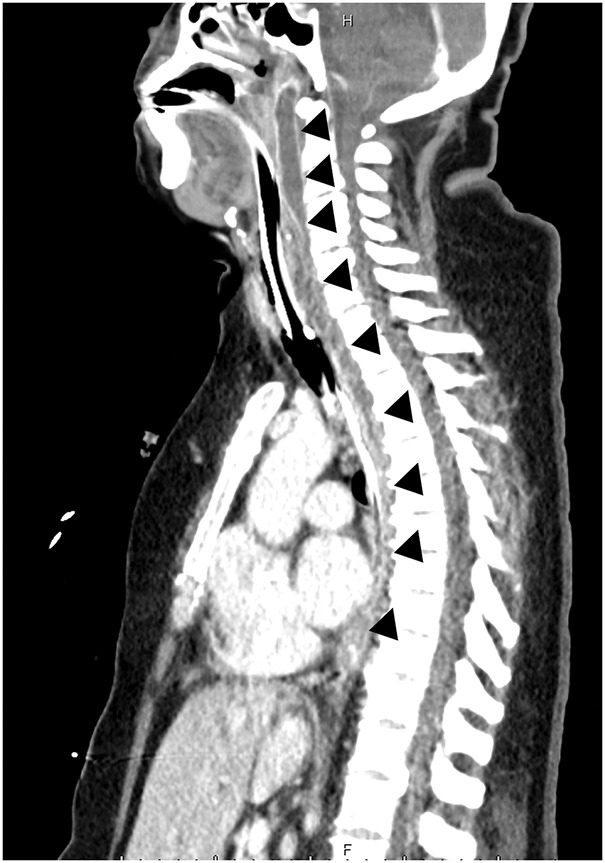
Enhanced CT scan of a sagittal plane before emergency surgery. Fluid collection with an enhanced wall, indicating an abscess in the hypopharynx extending to the mediastinum (black triangles).

Additional sagittal plane reconstruction revealed an abscess in the hypopharyngeal cavity and mediastinum (**Fig. 3**). *Streptococcus constellatus* was also cultured from a pleural effusion sample, leading to a diagnosis of Type II DNM. Immediate surgical drainage was performed. During surgery, a linear skin incision was made on the left anterior side of the neck to access and drain the abscess behind the sternocleidomastoid muscle. Postoperatively, a gastric drainage tube was placed from the neck to the inferior mediastinum along the space between the esophagus and the prevertebral fascia, and a chest drain tube was installed on the left side. Continuous saline injection through a gastric tube facilitated the removal of purulent waste fluid via the chest drain. Her cardiopulmonary condition improved significantly, and she recovered gradually without further complications. One month after surgery, the patient was discharged, and her weight was successfully reduced to a BMI of 27 kg/m^2^ 10 months after emergency surgery.

## DISCUSSION

The clinical diagnosis of DNM is often delayed and challenging because nonspecific symptoms do not manifest until clinical deterioration occurs. The diagnosis was confirmed using cervical–thoracic CT scans, necessitating the inclusion of the cervical region to ensure an accurate diagnosis.^3–5)^ In this case, the diagnosis of DNM was delayed because the initial suspicion was for the more common complication of staple line leakage, despite the patient presenting with atypical symptoms such as chest pain and a lack of typical CT findings, such as air bubbles or fluid collections at the staple line. However, for patients exhibiting chest pain after LSG, it is recommended to perform a CT scan from the cervical to the abdominal region, including sagittal plane reconstruction, to detect DNM. Although an undetectable minor staple leakage might be missed without typical CT findings, our patient’s improvement after surgical intervention ruled out this possibility. Additional abdominal interventions, such as laparotomy or upper gastrointestinal endoscopy, are required to rule out minor leakage.

A 36-French rubber calibration tube is commonly used as a guide during stomach dissection in LSG. Consistent stomach dissection is difficult to achieve without this calibration tube,^6)^ and we used the tube in all cases unless the patient had a latex allergy. This insertion has been reported to cause mucosal erosion and iatrogenic injury to the piriform fossa, esophagus, and stomach.^1)^ Our study could not determine the actual frequency of iatrogenic injuries. However, the injury may have been overlooked or underestimated. In our case, no calibration-related injuries were observed during or immediately after the surgery. However, minor mucosal injuries may occur, and diabetes can lead to pharyngeal infections.

It has been reported that 17%–21% of pharyngeal infections may progress to mediastinitis,^7)^ and the risk factors for this extension are older age (>55 years), presence of diabetes, and involvement of retropharyngeal spaces.^2)^ Our patient had 2 risk factors: diabetes and retropharyngeal space involvement. Once mediastinitis develops, the mortality rate can be as high as 40%.^3)^ Therefore, prevention and early detection are critical. Although the patient was asymptomatic post-LSG, patients with diabetes were at a risk of developing DNM caused by minor mucosal trauma from the calibration tube. This case highlights the importance of careful observation and anticipation of iatrogenic injury caused by calibration tube passage, especially in the pharynx, for safe LSG.

## CONCLUSIONS

To date, the diagnosis of DNM following LSG has not been well recognized, which has led to delays in diagnosis. However, we successfully managed the patient after the correct diagnosis was made. In this case, an unnoticed piriform fossa injury during LSG, potentially exacerbated by diabetes, led to a pharyngeal infection that resulted in deadly DNM. A comprehensive CT scan from the neck to the abdomen is essential for an accurate diagnosis when complications from LSG are suspected.

## ACKNOWLEDGMENTS

We thank the members of our department, especially Dr. Masaru Tsuchiya, Dr. Takashi Oshiro, and Dr. Munetaka Ushio, for providing advice during the emergency surgery.

## DECLARATIONS

### Funding

This study was not supported by any funding agency.

### Authors’ contributions

K.K. and K.W.: Conceptualized and wrote the manuscript.

K.W.: Edited the manuscript.

The final version of the manuscript has been read and approved by all the authors.

All authors agree to take responsibility for all aspects of this study.

### Availability of data and materials

The data supporting the findings of this study are available upon request from the corresponding author. The data are not publicly available due to concerns regarding patient privacy.

### Ethics approval and consent to participate

Ethical approval was obtained from the Institutional Ethics Committee (IRB#S18061). All procedures involving human participants were performed in accordance with the ethical standards of the Institutional Research Committee and the 1964 Declaration of Helsinki and its later amendments.

### Consent for publication

Informed consent for publication was obtained from the patient involved in this study.

### Competing interests

The authors declare that they have no conflicts of interest.
